# Low-molecular-weight heparin for the prevention of preeclampsia in high-risk pregnancies without thrombophilia: a systematic review and meta-analysis

**DOI:** 10.1186/s12884-023-06218-9

**Published:** 2024-01-17

**Authors:** Jiahui Chen, Jing Huai, Huixia Yang

**Affiliations:** 1 Department of Obstetrics and Gynecology, Peking University First Hospital, No. 1 Xi ’an Men Street, Xicheng District, Beijing, 100034 China; 2 Beijing Key Laboratory of Maternal Fetal Medicine of Gestational Diabetes Mellitus, Beijing, China

**Keywords:** Low molecular weight heparin, Preeclampsia, Thrombophilia, Low-dose aspirin

## Abstract

**Objectives:**

To systematically evaluate the efficacy of low molecular weight heparin (LMWH) to prevent preeclampsia in high risk pregnant women without thrombophilia.

**Search strategy:**

PubMed, Embase and the Cochrane library were searched for articles published before 1st August 2022 using the combination keywords “preeclampsia”, “Low Molecular Weight Heparin”, “LMWH”, “Heparin, Low Molecular Weight”, “Dalteparin”, “Nadroparin”, and “Tinzaparin”. Selection criteria: Randomized controlled trials evaluating the use of LMWH in pregnant women at high risk of preeclampsia without thrombophilia.

**Data collection and analysis:**

Ten studies were included in the meta-analysis (1758 patients in total). Outcomes were expressed as relative risk (RR) with 95% confidence intervals (CI).

**Results:**

LMWH reduced the incidence of PE (RR = 0.67; 95% CI = 0.50–0.90; P = 0.009) in high risk pregnant women without thrombophilia. Subgroup analysis found that the prophylactic effect of LMWH was only significant in studies using low-dose aspirin (LDA) as the primary intervention. The combination of LMWH and LDA was also effective for the prevention of preterm birth and fetal growth restriction, but had no effect on the incidence of placenta abruption.

**Conclusion:**

For women at high risk of developing preeclampsia without thrombophilia, the combination of LMWH and low-dose aspirin is effective for the prevention of preeclampsia, preterm birth and fetal growth restriction and is superior to LDA alone.

**Supplementary Information:**

The online version contains supplementary material available at 10.1186/s12884-023-06218-9.

## Introduction

Preeclampsia (PE) is defined as new onset hypertension present after 20 weeks of gestation combined with proteinuria (> 300 mg/day) or other maternal organ dysfunction, such as renal insufficiency, liver involvement, neurological or hematological complications, uteroplacental dysfunction, or fetal growth restriction (FGR) [1]. PE is the primary cause of preterm birth and maternal mortality and affects approximately 3–5% of pregnancies. The pathogenesis of PE is unclear [2]. Numerous researches have aimed to predict, prevent and treat PE, including finding biomarkers in plasma, lifestyle interventions and drug prophylaxis. However, the identification of biomarkers or the development of diagnostic tools to predict PE has proven of limited value [3]. Safe and effective intervention is lacking and remains to be fully investigated [4].

Low-molecular-weight heparin (LMWH) is an anticoagulant widely used to prevent blood clots and treat deep vein thrombosis, pulmonary embolism, and myocardial infarction. LMWH is also the most commonly recommended anticoagulant for both prophylaxis and treatment of thrombosis in pregnancy and has also proven effective and safe in multiple prospective clinical trials [5]. There is evidence suggesting that LMWH might prevent maternal disease through anticoagulation-independent mechanisms [6]. Clinical trials have also focused on the expanded use of LMWH for other diseases, including abortion and pregnancy [7]. Several studies have been performed to investigate whether LMWH prevents PE [3–5], but the current intervention strategy and effect of LMWH in the prevention of PE are controversial, and the mechanisms need to be clarified [7, 8].

Although models for PE prediction are still under investigation, we can distinguish between women who are at low risk and high risk. Pregnancies with previous PE or hypertension in pregnancy or other risk factors are at high risk of developing PE [2]. There is an increase in the relative risk of placenta mediated complications, including PE associated with thrombophilia, regardless of inheritance and acquisition [8]. Prophylactic- or intermediate-dose LMWH has already been recommended to pregnant women with thrombophilia and prior venous thrombus embolism (VTE) or a positive family history for VTE [9]. However, for women without thrombophilia who are at a high risk of placenta mediated complications, whether LMWH is useful is still under debate. Several clinical trials were conducted to investigate the effect of LMWH on pregnancies without thrombophilia [10, 11].

This systematic review and meta-analysis aimed to determine the efficacy of prophylactic use of LMWH for the prevention of PE in women at high risk of developing PE without thrombophilia.

## Materials and methods

### Source and search strategy

PubMed, Embase and the Cochrane library were searched from their inception until August 1st, 2022 using the combination keywords “preeclampsia”, “Low Molecular Weight Heparin”, “LMWH”, “Heparin, Low Molecular Weight”, “Dalteparin”, “Nadroparin”, and “Tinzaparin”. A hand search of the reference lists of the included articles, relevant meta-analyses and reviews was performed. “Gray literature” was not searched. We reviewed abstracts of congresses and scientific meetings, reference lists of retrieved articles, published study protocols, previously published systematic reviews, and review articles of all relevant studies. No language restriction was imposed.

### Study selection

Randomized controlled trials (RCTs) published full texted in peer-reviewed journals with impact factors that evaluated the use of LMWH in pregnant women at high risk of PE without thrombophilia were eligible to be included in this study. Trials assessing the role of LMWH in pregnant women with acquired or inherited thrombophilia or as prophylaxis for venous thromboembolic disease in pregnancy and the postpartum period and quasi-randomized trials (e.g., those randomized by date of birth or hospital number) were excluded. Non-RCTs or studies including women with thrombophilia were excluded from this review.

### Data extraction

The selection process and data extraction were performed independently using a standardized procedure by three reviewers (JC, JH, and ML). First author, year of publication, study design, type and dose of LMWH, intervention of the control group, number of participants, baseline characteristics of pregnant women and maternal and fetal outcomes were the data extracted from the included studies. Any discrepancy was resolved by the consensus of all authors. All identified RCTs were included irrespective of their risk of bias level. JC and JH assessed the risk of bias of the final included studies and performed data analysis.

### Risk of bias

The risk of bias in each included study was assessed using the criteria outlined in the Cochrane Handbook for Systematic Reviews of Interventions. Seven domains related to the risk of bias were assessed in each included trial since there is evidence that these issues are associated with biased estimates of treatment effect: (1) random sequence generation; (2) allocation concealment; (3) blinding of participants and personnel; (4) blinding of outcome assessment; (5) incomplete outcome data; (6) selective reporting; and (7) other bias [12].

All analyses were performed using an intention-to-treat approach, evaluating women according to the treatment group to which they were randomly allocated in the original trials. This review was registered with the PROSPERO International Prospective Register of Systematic Reviews (Registration Number: CRD42020207474).

### Outcomes

The primary outcome was the occurrence of PE. Secondary outcomes included maternal and fetal outcomes related to placental dysfunction including placenta abruption, preterm birth (less than 34 weeks’ gestation), and FGR.

### Statistical analysis

The data analysis was completed independently by two authors (JC, JH) using Review Manager software (RevMan 2014). The completed analyses were then compared, and any difference was resolved by discussion with a third reviewer (ML).

Data from each eligible study were extracted without modification of original data onto custom-made data collection forms. For continuous outcomes, the mean standard deviation was extracted and imported into RevMan 2014.

Meta-analysis was performed using the fixed effects model to produce summary treatment effects in either a relative risk (RR) or mean difference (MD) with a 95% confidence interval (CI). Heterogeneity was measured using I-squared (Higgins I^2^).

Potential publication biases were assessed statistically using Begg’s and Egger’s tests. A P value < 0.05 was considered statistically significant.

The meta-analysis was reported following the Preferred Reporting Item for Systematic Reviews and Meta-analyses (PRISMA) statement [13].

## Results

### Study selection and study characteristics

**Fig. 1:**
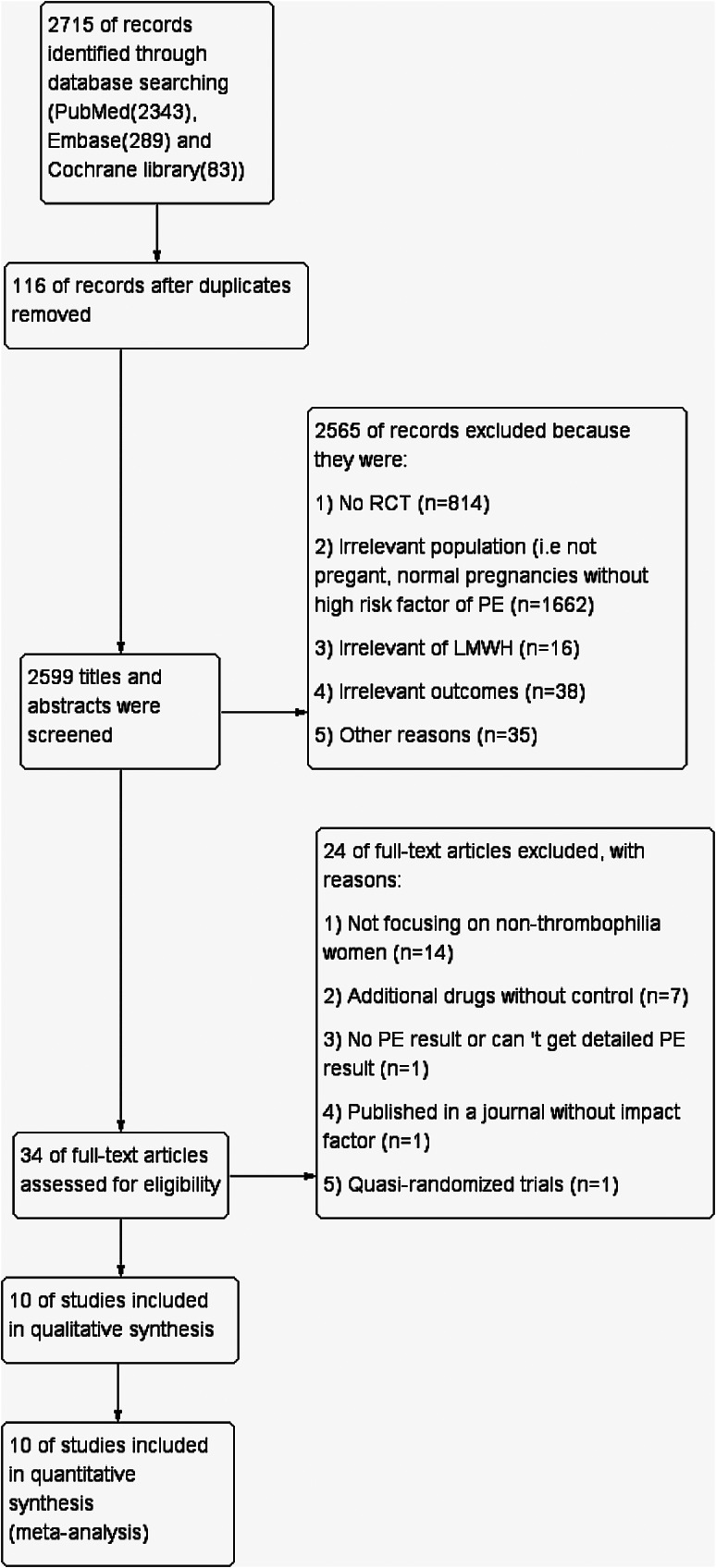
PRISMA flowchart of studies identified in the systematic review

The flow of study identification is shown in Fig. 1. Ten trials were identified as relevant and included in the meta-analysis [14–23]. In total, 1758 women at high risk of developing PE were included, of whom 906 were treated with a prophylactic daily dose of LMWH during pregnancy and 852 were treated with placebo or no treatment. Six articles used low-dose aspirin (LDA) as a basic intervention for both the experimental and control groups, four without LDA. The main characteristics of the ten included RCTs are presented in Table 1.

**Table 1:** Characteristics of the included trials

Reference	n	GA at recruitment(days)	Inclusion criteria (the criteria of high risk of preeclampsia)	Concomitant use of aspirin	Interventions (sample size)		Outcome
					Experiential group	Control group	
Fawzy et al. [14]	107	33–49	>= three documented IRM, no other reason for anticoagulation	No	Enoxaparin, 2000 IU daily (57)	Placebo (50)	Live births, preeclampsia, preterm delivery, gestational diabetes, and neonatal outcome
Rey et al. [15]	116	51–102	Complications in the immediate previous pregnancy without previous venous or arterial thrombotic event	The use of aspirin according to local standard practice for high-risk pregnancies at each center	Dalteparin, 4000 IU in women < 60 kg, 5000 IU in women 60–90 kg, 6000 IU in women > 90 kg (57) without/with Aspirin (51)	Aspirin (46) or not (11)	Severe PET, newborn weight < = the 5th percentile, major abruptio placentae and non-severe PET, newborn weight between the 6th and 10th percentile and gestational age at delivery
Gris et al. [16]	160	35–50	Abruptio placentae during the first pregnancy, without absolute indication for anticoagulant therapy	Aspirin 100 mg per day to pregnant women according to the usual local protocol	Enoxaparin, 4000 IU daily (80) without/with Aspirin 100 mg daily (15)	Aspirin 100 mg daily (33) or not (47)	preeclampsia, IUGR restricted to newborn birthweight ≤ the 5th percentile, abruptio placenta
Gris et al. [17]	224	34–50	Severe PE during the first pregnancy, without absolute indication for anticoagulant therapy	Aspirin 100 mg per day to all women	Enoxaparin, 4000 IU (112) with Aspirin 100 mg daily	Aspirin 100 mg daily (112)	PE, SGA restricted to newborn birthweight ≤ 5th percentile, placenta abruption, IUFD after 20 weeks of gestation
Martinelli et al. [18]	135	63–91	History of placenta complications in previous pregnancy without absolute indication for anticoagulant therapy.	No	Nadroparin, 3800 IU (63)	Medical surveillance (65)	Preeclampsia, eclampsia, HELLP syndrome, intrauterine fetal death, FGR, or placental abruption.
Pasquier et al. [19]	258	29–49	History of unexplained recurrent miscarriage, without antiphospholipid syndrome and inherited thrombophilia.	No	Enoxaparin, 4000 IU (92)	Standard care and pregnancy monitoring (88)	Intrauterine fetal death, preeclampsia, birth of a small-for-gestational-age neonate, placental abruption, and premature delivery.
Haddad et al. [20]	244	72–89	A confirmed history of previous severe preeclampsia, without anticoagulants judged by the local investigator	Aspirin 100 mg per day to all women	Enoxaparin, 4000 IU (122) with Aspirin 100 mg daily	Aspirin 100 mg daily (122)	Maternal death, perinatal death, preeclampsia, small for gestational age (less than the 10th percentile), and placental abruption
Groom et al. [21]	149	42–111	At risk of preeclampsia and/or IUGR based on their obstetric history, without previous thrombosis or APS.	Aspirin 100 mg per day to all women	Enoxaparin, 4000 IU (72) with Aspirin 100 mg daily	Aspirin 100 mg daily (77)	Preeclampsia and/or SGA.
Shaaban et al. [22]	300	42	3 or more spontaneous consecutive miscarriages before 20 weeks’ gestation, without any known cause of recurrent miscarriage	No	Tinzaparin sodium, 0.4 mg/kg (109)	No placebo (69)	Take-home baby rate, miscarriage rate, occurrence of pregnancy complications such as IUGR or preeclampsia.
Llurba et al. [23]	278	60–110	Severe PE or IUGR in previous pregnancy or positive first trimester screening for PE	Aspirin 100 mg per day to women with prior early-onset PE	Enoxaparin, 4000 IU daily (144) without/with Aspirin 100 mg daily (20)	Aspirin 100 mg daily (26) or not (108)	

The quality of the RCTs included in our meta-analysis was assessed using the seven criteria outlined in the Cochrane Handbook for Systematic Reviews of Interventions. Most of the included studies were judged as “low risk” of bias in most of the seven Cochrane domains related to the risk of bias (Supplemental Material). All the included studies had a “low risk” of bias in “random sequence generation”. All but one (Pasquier2015) of the included studies had a “high risk” of bias in “blinding of participants and personnel (performance bias)” since the daily subcutaneous injection of LMWH was challenging to be blinded. Methodological quality assessment for each individual study, risk of bias graph, risk of bias graph summary, and publication bias funnel plot can be found in the Supplemental Material.

Publication bias, assessed statistically using Begg’s and Egger’s tests, showed no significant bias (P = 0.533 and P = 0.353, respectively). Statistical heterogeneity within the trials ranged from low to moderate with no inconsistency (I^2^ = 0%) for several secondary outcomes and I^2^ = 38% for the primary outcome.

### Meta-analysis results

All ten trials only included pregnant women at high risk of developing PE. All the articles excluded women with thrombophilia and reported the incidence of PE.

**Fig. 2:**
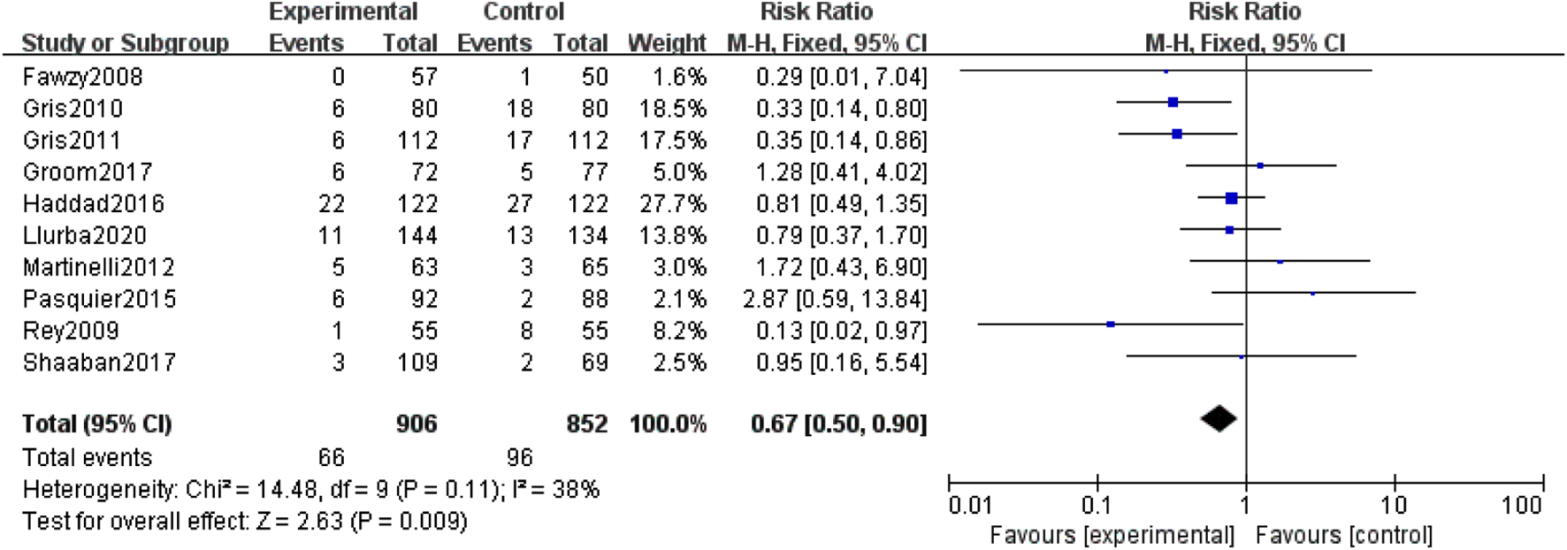
Forest plot for the relief influence of LMWH on PE

**Fig. 3:**
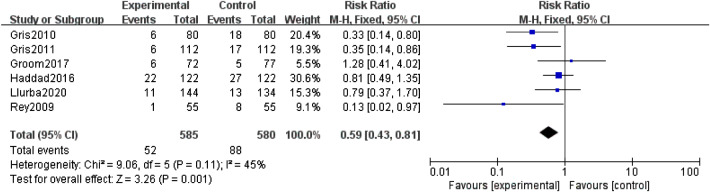
Forest plot for the relief influence of LMWH on PE in studies with LDA

**Fig. 4:**
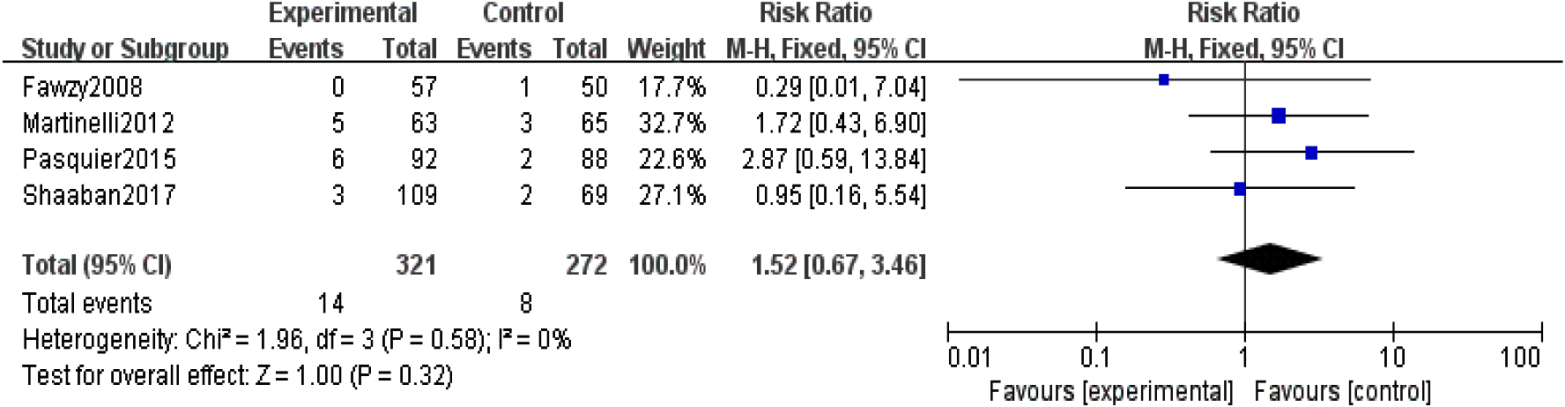
Forest plot for the relief influence of LMWH on PE in studies without LDA

The primary outcome was analysed with the fixed-effect model, and the pooled estimate of the ten included RCTs suggested that compared to the control group, prophylactic use of LMWH showed a relief influence on PE (RR = 0.67; 95% CI = 0.50–0.90; P = 0.009), with no significant heterogeneity among the studies (I^2^ = 38%, heterogeneity P = 0.11, Fig. 2). In the subgroup analysis, the prophylactic effect of LMWH was only significant in studies that used LDA as the primary intervention (RR = 0.59; 95% CI = 0.43–0.81; P = 0.001, Fig. 3), while the result was not significant in studies that did not use LDA (RR = 1.52; 95% CI = 0.67–3.46; P = 0.32, Fig. 4).

Other results, including preterm birth, IUGR and placenta abruption, are shown in Table 2. In the secondary outcome analysis, the prophylactic effect of LMWH was also significant on preterm birth and FGR, of which the effect was only significant in studies using LDA as the primary intervention. Data from the 10 included trials showed that patients treated with LMWH presented significantly fewer preterm birth than those not treated with LMWH (RR = 0.62; 95% CI = 0.46–0.84; P = 0.002) on the premise of the primary intervention of LDA. Patients treated with LMWH presented significantly fewer fetal growth restriction than those not treated with LMWH (RR = 0.71; 95% CI = 0.55–0.91; P = 0.007) on the premise of the primary intervention of LDA. No statistically significant differences were found in the occurrence of placental abruption between LMWH-treated and nontreated patients regardless of whether the LDA is used as a primary intervention.

**Table 2:** Relative risk of placenta-mediated complications in studies with and without LDA

	Trials, n	Participants, n	Random effect, Relative risk (95% CI)	P value	I^2^, %
Preeclampsia	10	1758	0.67 (0.50–0.90)	0.009	38
With LDA as the primary intervention	6	1165	0.59 (0.43–0.81)	0.001	45
Without LDA as the primary intervention	4	593	1.52 (0.67–3.46)	0.32	0
Preterm birth	10	1758	0.63 (0.48–0.83)	0.001	0
With LDA as the primary intervention	6	1165	0.62 (0.46–0.84)	0.002	0
Without LDA as the primary intervention	4	593	0.69 (0.33–1.47)	0.34	0
Fetal growth restriction	10	1758	0.72 (0.56–0.91)	0.007	44
With LDA as the primary intervention	6	1165	0.71 (0.55–0.91)	0.007	59
Without LDA as the primary intervention	4	593	0.86 (0.32–2.30)	0.76	1
Placental abruption	6	1046	0.50 (0.19–1.33)	0.16	0
With LDA as the primary intervention	4	738	0.52 (0.19–1.46)	0.21	0
Without LDA as the primary intervention	2	308	0.34 (0.01–8.28)	0.51	-

Data are reported as relative risk and 95% confidence interval in studies with LDA and without LDA. I^2^: heterogeneity; CI, confidence interval

## Discussion

### Main findings

All ten studies in this meta-analysis only included pregnancies with a high risk of developing PE without thrombophilia. The findings of this systematic review and meta-analysis suggest that in women at high risk of developing PE without thrombophilia, the combination of LMWH and low‐dose aspirin is effective for the prevention of PE, preterm birth and FGR and is superior to LDA alone.

In studies that did not use aspirin as a baseline intervention, LMWH showed no prophylaxis effect for PE [14, 18, 19, 22]. A possible explanation for this phenomenon we suspect is that pregnant women who need to use LDA are more susceptible to thrombotic events and placental complications such as PE. Another possible explanation is that LMWH needs to work on the effect of aspirin. Since preterm birth and FGR are the pregnancy comorbidities associated with PE [24], the preventive effect of LMWH to preterm birth and FGR may be due to its preventive role in PE, or it may be that LMWH can acting on the pathogenesis of preterm birth and FGR.

### Comparison of results with those of previous studies

We observed that the benefits of LMWH to PE are limited to the combination of LDA. A meta-analysis including 403 participants that evaluated the effect of treatment with LMWH for the prevention of PE in non-thrombophilic women found that the general use of LMWH was associated with a risk reduction for PE [10]. However, it did not compare the effect of LMWH separately from LDA. Some studies have explored the effect of LMWH on PE based on aspirin and found that treatment with LMWH and aspirin, compared with aspirin alone, was associated with a significant reduction in PE [25–27], while some of them have found that treatment with LMWH has no significant benefit in preventing PE [28].

The strength of our study is that the thrombophilia population was excluded from the included population. Another advantage of our study is that we explored the role of LMWH in the prevention of preeclampsia with or without LDA as a primary treatment. Based on the results of the above trials and our meta-analysis, it could be suggested that in high‐risk pregnancies, the prophylactic use of LMWH with the combination of LDA is beneficial in reducing PE, preterm birth and FGR in high-risk pregnancies without thrombophilia.

### Limitations of the study

While all ten studies included in our study considered medical history as the main risk factor for PE, we did not explore the preventive effect of LMWH for PE in other high risk populations, such as obese pregnant women. Other risk factors from maternal demographic characteristics also need to be identified. As an increasing number of RCT studies incorporate the demographic characteristics and medical history of pregnant women into the risk factors for PE, future studies are suspected to cover the preventive effect of LMWH for PE in different high risk populations.

Another limitation of our analysis relates to the small number of studies in the subgroup analysis of LDA use and no-LDA use. Consequently, the possibility of bias in the results could not be assessed by funnel plot and sensitivity analysis, and therefore we cannot exclude the possibility of publication bias. Other limitations include, based on the negative effect of no-LDA use, the effect of other subgroups’ proceeding analysis was insignificant, such as subgroups of different kinds of LMWH.

### Electronic supplementary material

Below is the link to the electronic supplementary material.

**Supplementary Material 1:** Methodological Quality Assessment

## Data Availability

All authors had full access to the data and materials. Data is available from the authors upon reasonable request.

## Declarations

### Statement

The manuscript has been read and approved by all the authors.
