# Supplementary material for: Low-molecular-weight heparin for the prevention of preeclampsia in high-risk pregnancies without thrombophilia: a systematic review and meta-analysis
Source: BMC Pregnancy Childbirth. 2024 Jan 17;24:68. doi: 10.1186/s12884-023-06218-9 (PMC10792962; doi:10.1186/s12884-023-06218-9)

Supplemental material

Methodological Quality Assessment

Studies included in the present metaanalysis are shown below in alphabetical order, with their respective methodological quality assessment.

1. Fawzy M, Shokeir T, El-Tatongy M, Warda O, El-Refaiey AAA, Mosbah A. Treatment options and pregnancy outcome in women with idiopathic recurrent miscarriage: A randomized placebo-controlled study. Archives of Gynecology and Obstetrics 2008; 278(1): 33-8.

Study: Fawzy et al., 2008.

Domain	Risk of bias	Support of judgment
Random sequence generation (selection bias)	Low	Method of randomisation: computer-generated list of study numbers.
Allocation concealment (selection bias)	Low	Allocation concealment: patients were blinded to the treatment allocation.
Blinding of participants and personnel (performance bias)	High	No.
Blinding of outcome assessment (detection bias)	Uncertain	Not stated.
Incomplete outcome data (attrition bias)	Low	Six patients were lost to follow-up (2 from enoxaparin arm and 4 from combination treatment arm) and four women stopped treatment (1 from enoxaparin arm and 3 from combination treatment arm).
Selective reporting (reporting bias)	Uncertain	Unable to assess.
Other biases	Low	No other potential bias identified.
Global assessment: Uncertain.		

2. Rey E, Garneau P, David M, et al. Dalteparin for the prevention of recurrence of placental-mediated complications of pregnancy in women without thrombophilia: A pilot randomized controlled trial. Journal of Thrombosis and Haemostasis 2009; 7(1): 58-64.

Study: Rey et al., 2009.

Domain	Risk of bias	Support of judgment
Random sequence generation (selection bias)	Low	Method of randomisation: a computer generated random numbers table (blocks of six).
Allocation concealment (selection bias)	Low	Allocation concealment: sealed opaque envelope containing the name of the group to which the woman was randomized .
Blinding of participants and personnel (performance bias)	High	No.
Blinding of outcome assessment (detection bias)	Uncertain	Blinding of outcome assessors: two adjudicators blinded to treatment assignment and not involved in the study reviewed all the patient report forms.
Incomplete outcome data (attrition bias)	Low	Data from all women were analyzed in their respective group of randomization in the intention-to-treat analysis.
Selective reporting (reporting bias)	Uncertain	Unable to assess.
Other biases	Low	No other potential bias identified.
Global assessment: Uncertain.		

3. Gris JC, Chauleur C, Faillie JL, et al. Enoxaparin for the secondary prevention of placental vascular complications in women with abruptio placentae. The pilot randomised controlled NOH-AP trial. Thromb Haemost 2010; 104(4): 771 - 9.

Study: Gris et al., 2010.

Domain	Risk of bias	Support of judgment
Random sequence generation (selection bias)	Low	Method of randomisation: computer-generated random numbers table (blocks of six).
Allocation concealment (selection bias)	Low	Allocation concealment: sealed opaque envelopes.
Blinding of participants and personnel (performance bias)	High	No.
Blinding of outcome assessment (detection bias)	Uncertain	Blinding of outcome assessors: three adjudicators blinded to treatments.
Incomplete outcome data (attrition bias)	Low	7 censored data were also included in the analysis.
Selective reporting (reporting bias)	Uncertain	Unable to assess.
Other biases	Low	No other potential bias identified.
Global assessment: Uncertain.		

4. Gris JC, Chauleur C, Molinari N, et al. Addition of enoxaparin to aspirin for the secondary prevention of placental vascular complications in women with severe pre-eclampsia. The pilot randomised controlled NOH-PE trial. Thromb Haemost 2011; 106(6): 1053 - 61.

Study: Gris et al., 2011.

Domain	Risk of bias	Support of judgment
Random sequence generation (selection bias)	Low	Method of randomisation: computer-generated random numbers table (blocks of six).
Allocation concealment (selection bias)	Low	Allocation concealment: sealed opaque envelopes.
Blinding of participants and personnel (performance bias)	High	No.
Blinding of outcome assessment (detection bias)	Uncertain	Blinding of outcome assessors: three adjudicators blinded to treatments.
Incomplete outcome data (attrition bias)	Low	18 censored data were also included in the analysis.
Selective reporting (reporting bias)	Uncertain	Unable to assess.
Other biases	Low	No other potential bias identified.
Global assessment: Uncertain.		

5. Martinelli I, Ruggenenti P, Cetin I, et al. Heparin in pregnant women with previous placenta-mediated pregnancy complications: a prospective, randomized, multicenter, controlled clinical trial. Blood 2012; 119(14): 3269 - 75.

Study: Martinelli et al., 2012.

Domain	Risk of bias	Support of judgment
Random sequence generation (selection bias)	Low	Method of randomisation: A computer randomization list.
Allocation concealment (selection bias)	Low	Allocation concealment: The patient randomization number was requested by phone or fax and centrally assigned by the treatment secretariat.
Blinding of participants and personnel (performance bias)	High	No.
Blinding of outcome assessment (detection bias)	Uncertain	Blinding of outcome assessors: independent adjudicator (P.R.) who was blinded to treatment allocation.
Incomplete outcome data (attrition bias)	Low	7 drop-outs with no influence to the outcome analysis.
Selective reporting (reporting bias)	Uncertain	Unable to assess.
Other biases	Low	No other potential bias identified.
Global assessment: Uncertain.		

6. Pasquier E, de Saint Martin L, Bohec C, et al. Enoxaparin for prevention of unexplained recurrent miscarriage: a multicenter randomized double-blind placebo-controlled trial. Blood 2015; 125(14): 2200‐5.

Study: Pasquier et al., 2015.

Domain	Risk of bias	Support of judgment
Random sequence generation (selection bias)	Low	Method of randomisation: a central web-based randomization system.
Allocation concealment (selection bias)	Low	Allocation concealment: blocked randomization (allocation ratio of 1:1, block size of 6) was stratified according to study center and to 3 levels of disease severity, based on combination of woman's age and the number of previous miscarriages.
Blinding of participants and personnel (performance bias)	High	Blinding of participants and caregivers: packed in identical sachets.
Blinding of outcome assessment (detection bias)	Uncertain	Blinding of outcome assessors: unaware of the study group assignments.
Incomplete outcome data (attrition bias)	Low	All women underwent randomization were included in intention-to-treat analysis.
Selective reporting (reporting bias)	Uncertain	Unable to assess.
Other biases	Low	No other potential bias identified.

Global assessment: Uncertain.

7. Haddad B, Winer N, Chitrit Y, et al. Enoxaparin and Aspirin Compared with Aspirin Alone to Prevent Placenta-Mediated Pregnancy Complications. Obstetrics and Gynecology 2016; 128(5): 1053-63.

Study: Haddad et al., 2016.

Domain	Risk of bias	Support of judgment
Random sequence generation (selection bias)	Low	Method of randomisation: computer-based randomization list.
Allocation concealment (selection bias)	Low	Allocation concealment: treatment allocation in the presence of the participant .
Blinding of participants and personnel (performance bias)	High	No.
Blinding of outcome assessment (detection bias)	Uncertain	Outcome adjudicators were masked to treatment assignment.
Incomplete outcome data (attrition bias)	Low	Patient lost to follow with unavailable outcome data were included in the intention to treat analysis.
Selective reporting (reporting bias)	Uncertain	Unable to assess.
Other biases	Low	No other potential bias identified.

Global assessment: Uncertain.

8. Groom KM, McCowan LM, Mackay LK, et al. Enoxaparin for the prevention of preeclampsia and intrauterine growth restriction in women with a history: a randomized trial. American journal of obstetrics and gynecology 2017; 216(3): 296.e1-.e14.

Study: Groom et al., 2017.

Domain	Risk of bias	Support of judgment
Random sequence generation (selection bias)	Low	Method of randomisation: computer-generated randomization program balanced in blocks of 5.
Allocation concealment (selection bias)	Low	Allocation concealment: sealed opaque envelopes.
Blinding of participants and personnel (performance bias)	High	No.
Blinding of outcome assessment (detection bias)	Uncertain	No.
Incomplete outcome data (attrition bias)	Low	Women miscarriage <16 weeks and discontinued intervention were also included in the analysis.
Selective reporting (reporting bias)	Uncertain	Unable to assess.
Other biases	Low	No other potential bias identified.
Global assessment: Uncertain.		

9. Shaaban OM, Abbas AM, Zahran KM, Fathalla MM, Anan MA, Salman SA. Low-Molecular-Weight Heparin for the Treatment of Unexplained Recurrent Miscarriage with Negative Antiphospholipid Antibodies: A Randomized Controlled Trial. Clinical and Applied Thrombosis/Hemostasis 2017; 23(6): 567-72.

Study: Shaaban et al., 2017.

Domain	Risk of bias	Support of judgment
Random sequence generation (selection bias)	Low	Method of randomisation: a computer-generated random table.
Allocation concealment (selection bias)	Low	Allocation concealment: scertained using serially numbered closed opaque envelopes.
Blinding of participants and personnel (performance bias)	High	No.
Blinding of outcome assessment (detection bias)	Uncertain	Not stated.
Incomplete outcome data (attrition bias)	Low	All women underwent randomizatin were included in the analysis.
Selective reporting (reporting bias)	Uncertain	Unable to assess.
Other biases	Low	No other potential bias identified.
Global assessment: Uncertain.		

10. Llurba E, Bella M, Burgos J, et al. Early Prophylactic Enoxaparin for the Prevention of Preeclampsia and Intrauterine Growth Restriction: A Randomized Trial. Fetal Diagn Ther. 2020;47(11):824-833.

Study: Llurba et al., 2020.

Domain	Risk of bias	Support of judgment
Random sequence generation (selection bias)	Low	computer-generated allocation sequence (1:1 ratio)
Allocation concealment (selection bias)	Low	Not said.
Blinding of participants and personnel (performance bias)	High	No.
Blinding of outcome assessment (detection bias)	Uncertain	No.
Incomplete outcome data (attrition bias)	Low	Patient lost to follow with unavailable outcome data were included in the intention to treat analysis.
Selective reporting (reporting bias)	Uncertain	Not said.
Other biases	Low	Not said.
Global assessment: Uncertain.		

Risk of bias graph. Risk of bias items presented as percentages across all included studies

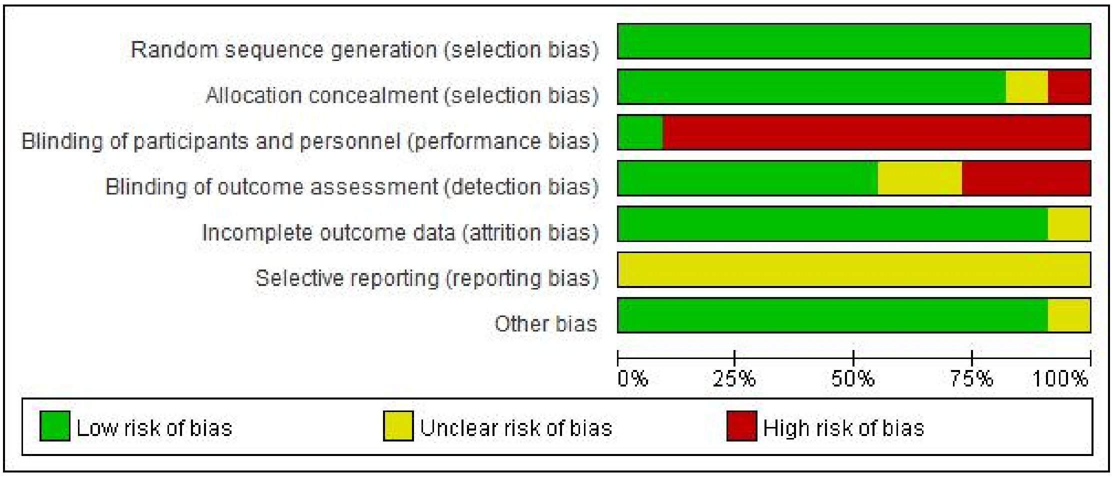

Risk of bias summary. Risk of bias items for each included study

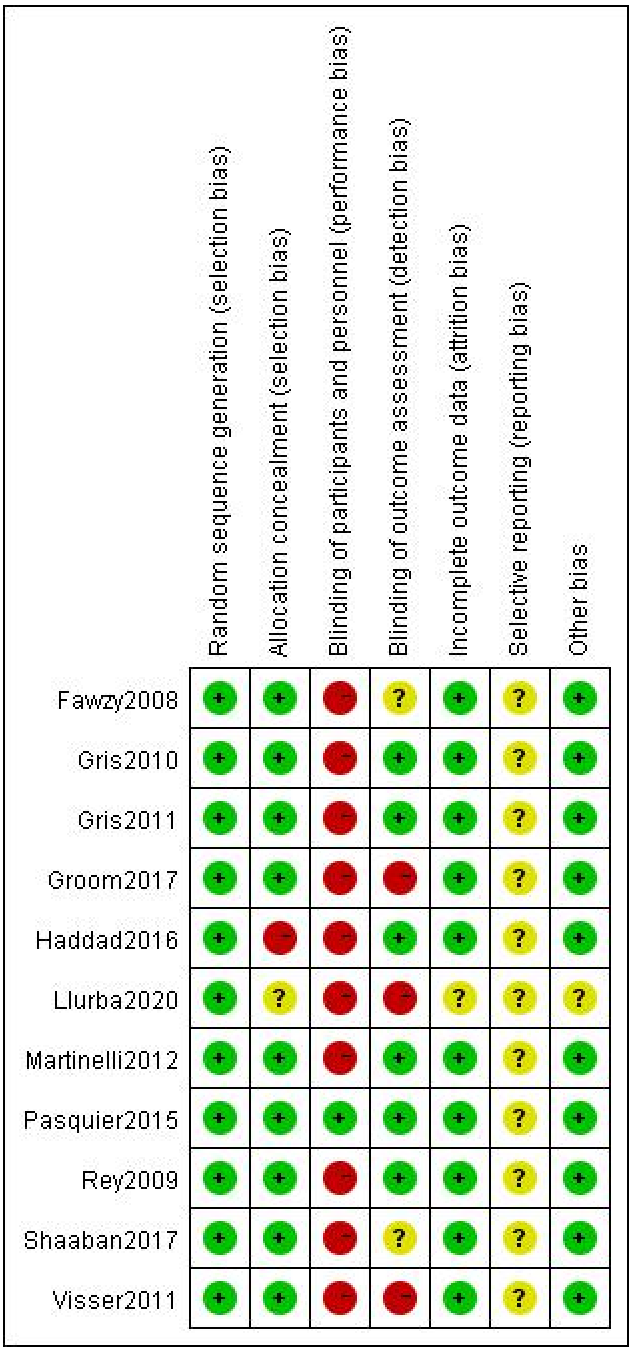

Publication bias. Funnel plot comparing low-molecular weight heparin for development of preeclampsia. The graph, which includes all 10 studies, showed no publication biases

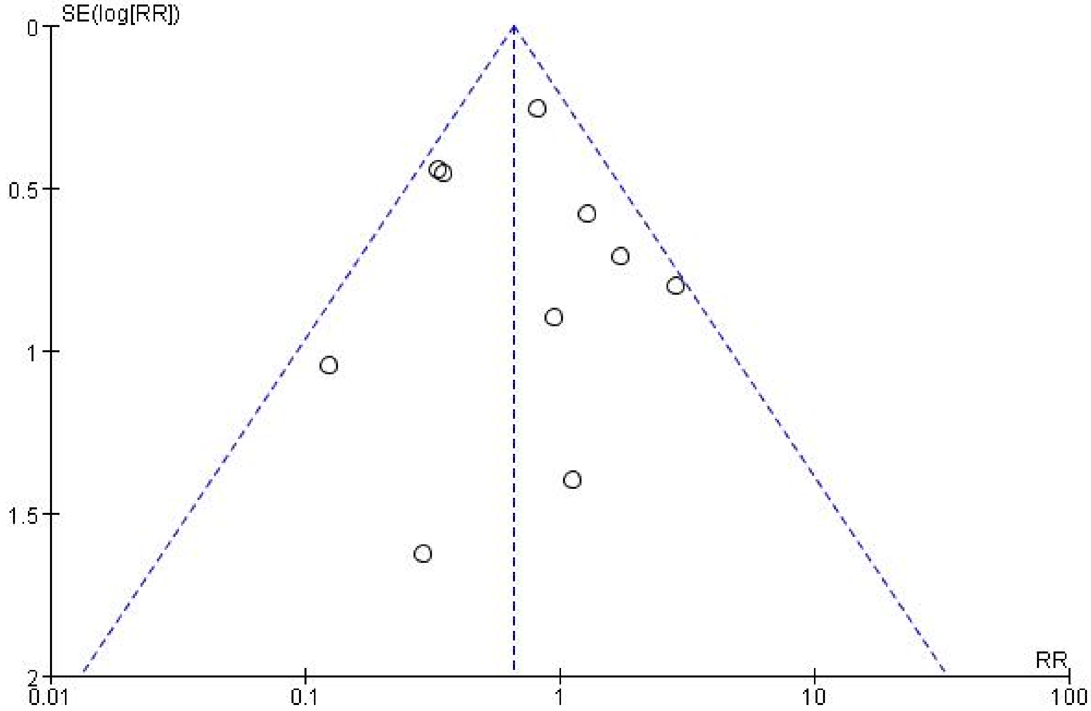

Supplement: Supplementary file 1 — Supplementary Material 1: Methodological Quality Assessment [file 12884_2023_6218_MOESM1_ESM.pdf]
